# The relationship between complement factor C3, *APOE* ε4, amyloid and tau in Alzheimer’s disease

**DOI:** 10.1186/s40478-016-0339-y

**Published:** 2016-06-29

**Authors:** Luke W. Bonham, Rahul S. Desikan, Jennifer S. Yokoyama, for the Alzheimer’s Disease Neuroimaging Initiative

**Affiliations:** Memory and Aging Center, Department of Neurology, University of California, San Francisco, 675 Nelson Rising Lane, Suite 190, San Francisco, CA 94158 USA; Neuroradiology Section, Department of Radiology and Biomedical Imaging, University of California, San Francisco, 505 Parnassus Avenue, San Francisco, 94143 CA USA

**Keywords:** Complement 3, Amyloid, Aβ, phospho-tau, ptau, APOE ε4, CSF, Cerebrospinal fluid, Alzheimer's disease

## Abstract

Inflammation is becoming increasingly recognized as an important contributor to Alzheimer’s disease (AD) pathogenesis. As a part of the innate immune system, the complement cascade enhances the body’s ability to destroy and remove pathogens and has recently been shown to influence Alzheimer’s associated amyloid and tau pathology. However, little is known in humans about the effects of the complement system and genetic modifiers of AD risk like the ε4 allele of apolioprotein E (*APOE* ε4) on AD pathobiology. We evaluated cerebrospinal fluid (CSF) protein levels from 267 individuals clinically diagnosed as cognitively normal, mild cognitive impairment, and AD. Using linear models, we assessed the relationship between *APOE* ε4 genotype, CSF Complement 3 (C3), CSF amyloid-β (amyloid) and CSF hyperphosphorylated tau (ptau). We found a significant interaction between *APOE* ε4 and CSF C3 on both CSF amyloid and CSF ptau. We also found that CSF C3 is only associated with CSF ptau after accounting for CSF amyloid. Our results support a conceptual model of the AD pathogenic cascade where a synergistic relationship between the complement cascade (C3) and *APOE* ε4 results in elevated Alzheimer’s neurodegeneration and in turn, amyloid further regulates the effect of the complement cascade on downstream tau pathology.

**Electronic supplementary material:**

The online version of this article (doi:10.1186/s40478-016-0339-y) contains supplementary material, which is available to authorized users.

## Introduction

Converging experimental and human biomarker evidence suggests that the immune system plays a critical role in Alzheimer’s disease (AD) pathobiology. In rodent models of AD, neuroinflammatory signals have been shown to correlate with amyloid and hyperphosphorylated tau (ptau) deposits as well as cognitive symptoms [23]. Observational studies in humans have shown that neuroinflammation, particularly the innate immune system, contributes to and drives AD pathogenesis [14]. Genetic studies in humans have identified a relationship between inflammatory and immune-associated genes and increased risk for AD [18] and have suggested that immune-mediated genes may contribute to Alzheimer’s pathogenesis [32]. Importantly, several studies have demonstrated that the ε4 allele of apolipoprotein (*APOE* ε4), the most common genetic risk factor for AD, has an important role in immune and inflammatory processes underlying Alzheimer’s neurodegeneration [6, 29]. Further, a recent study of epigenomic signals in mice and humans has implicated regulatory changes in immune response genes and immune-regulatory genes in AD [13].

Recent work suggests an association between inflammatory pathways, amyloid, and tau pathology. In mouse models, Complement C1q has been shown to be necessary for amyloid-β associated synaptotoxicity [15]. Using microarray data from amyloid and tau transgenic mice, immune gene expression has been shown to be tightly associated with amyloid plaques rather than neurofibrillary tangles suggesting that amyloid dysmetabolism mediates the influence of the immune system on tau pathology [19]. Although several studies have examined the association between inflammation and AD neurodegeneration in humans, it is still unknown whether *APOE* ε4 influences the relationship between inflammation (specifically the complement cascade) and amyloid and tau pathology.

Here, we investigated the relationship between *APOE* ε4 and the complement system on amyloid and tau pathology in AD. We evaluated cerebrospinal fluid (CSF) measurements of the immune protein Complement 3 (C3) as well as amyloid β_1-42_ (amyloid) and ptau_181p_ (ptau), which are sensitive *in vivo* markers of clinical diagnosis, decline, and pathology in AD [8, 9, 24]. Low CSF amyloid levels are associated with increased amyloid plaques in the brain while high CSF ptau levels are associated with increased ptau tangles in the brain [4, 9]. Low CSF C3 has been associated with worsening cognitive decline in mild cognitive impairment (MCI) [28] and improves diagnostic accuracy when used alongside CSF amyloid and tau [16]. We hypothesized that a statistical interaction between *APOE* ε4 and CSF C3 levels would be associated with low CSF amyloid (suggesting increased intracranial amyloid plaques) and elevated CSF ptau (suggesting increased intracranial ptau tangles). Further, building on prior work [15, 19], in a mediation analysis, we hypothesized that CSF amyloid would ‘mediate’ the effect of CSF C3 on CSF ptau.

## Materials and methods

### Participant description

This study utilized samples from cognitively normal older adults (HC; *n* = 71), individuals diagnosed with amnestic MCI (*n* = 110), and probable AD (*n* = 56) from the Alzheimer’s Disease Neuroimaging Initiative (ADNI), which has been used in previously published studies [10, 11]. Clinical severity of symptoms in the MCI and AD groupings was measured using the Clinical Dementia Rating Sum of Boxes (CDR-SB) [22] and Mini Mental State Exam (MMSE) [12]. Diagnostic groupings were assigned using the clinical judgment of each site’s clinicians along with cutoffs based on neuropsychological tests [33]. Briefly, controls were required to have normal memory function on the Logical Memory II subscale of the Weschler Memory Scale – Revised [31], an MMSE score greater than 24, CDR total score equal to 0, and judgment by a clinician that the individual did not have any significant impairment in cognitive function or activities of daily living. Individuals with MCI were required to have abnormal memory function on the Logical Memory II subscale of the Weschler Memory Scale – Revised, an MMSE greater than 24, CDR total score equal to 0.5, and judgment by a clinician that the individual’s general cognition and functional performance was preserved enough that a diagnosis of AD could not be made. Finally, individuals with AD were required to have abnormal memory function on the Logical Memory II subscale of the Weschler Memory Scale – Revised, an MMSE between 20 and 26, CDR total score equal to 0.5 or 1.0, and judgment by a clinician that the individual met NINCDS/ADRDA criteria for probable AD [20]. Informed consent was obtained from all individual participants included in the study. For more information on the ADNI cohort, see Additional file 1.

### Biomarker measurements

CSF amyloid β_1-42_ and CSF ptau_181p_ were measured using the AlzBio3 Luminex xMAP immunoassay (Innogenetics, Ghent, Belgium) according to previously described methods [24]. This method utilizes monoclonal antibodies specific for amyloid β_1-42_ and ptau_181p_ that are chemically bonded to color-coded beads along with analyte-specific detector antibodies. Baseline CSF C3 levels were measured using a separate multiplex immunoassay panel (Luminex technology), developed by Rules Based Medicine (Myriad RBM; Austin, Texas). CSF measurements in the immunoassay panel were processed and normalized according to previously described methods [7, 26]. Briefly, C3 levels were measured using the Myriad RBM Human DiscoveryMAP panel, which measures CSF protein levels for a range of metabolic, lipid, inflammatory, and other AD-relevant indicators. Myriad RBM used a Luminex 100 instrument for the measurements and analyzed the resulting data using proprietary software. The ADNI staff checked the distributions of analytes in this panel for normality by using Box-Cox analyses, and when appropriate log_10_ transformed the data to achieve an approximately normal distribution. *APOE* genotype was previously generated by ADNI using DNA extracted by Cogenics (now Beckman Coulter Inc.; Pasadena, California) from a 3 mL aliquot of EDTA blood. *APOE* ε4 dosage was scored as count of ε4 allele (0, 1, or 2) in all analyses.

### Statistical analysis

We used chi-squared and one-way ANOVA for demographic and biomarker summary statistics and univariate assessments. Multivariate linear models were used to assess all biomarker relationships, covarying for baseline age, baseline CDR-SB, and sex.

We first tested for a statistical interaction between CSF C3 levels and *APOE* ε4 dosage on CSF amyloid and CSF ptau levels. Prior to our secondary analyses, we verified that there was an association between CSF amyloid and CSF ptau levels.

Next, we evaluated two theoretical models by which CSF C3 levels might modify CSF ptau levels. In Model A, we tested whether CSF amyloid levels ‘mediate’ CSF ptau levels by adding it to a baseline model in which CSF C3 levels predict CSF ptau levels. In Model B, we test whether a statistical interaction between CSF C3 levels and CSF amyloid levels significantly predict CSF ptau levels.

All analyses were performed using R. Plots were made using the ‘visreg’ package in R [5].

## Results

### Cohort description

Our cohort was balanced with respect to age, sex, and CSF C3 levels as there were no significant differences by diagnostic group (Table 1). There were significant differences in baseline CDR-SB scores, MMSE scores, *APOE* ε4 dosage, CSF amyloid, and CSF ptau.

**Table 1:** Demographic, genetic, and biomarker data summarized by diagnostic group

	NC	MCI	AD	
N	71	110	56	*P*-value
Age (Mean ± SE)	76.5 ± 0.7	75.6 ± 0.7	74.9 ± 1.0	0.19
Sex (M/F)	36/35	72/38	30/26	0.11
CDR-SB (Mean ± SE)	0.04 ± 0.02	1.55 ± 0.09	4.13 ± 0.20	<0.001
*APOE* ε4 Dose (0/1/2)	56/14/1	47/49/14	17/25/14	<0.001
CSF amyloid β_1-42_ (Mean ± SE)	209.2 ± 6.2	158.6 ± 4.7	139.3 ± 4.4	<0.001
CSF ptau_181p_ (Mean ± SE)	25.5 ± 1.7	36.2 ± 1.5	43.0 ± 2.6	<0.001
CSF C3 (Mean ± SE)	−2.56 ± 0.02	−2.57 ± 0.02	−2.58 ± 0.02	0.53

*SE* standard error, *M* male, *F* female, *CDR-SB* clinical dementia rating sum of boxes score, *MMSE* mini mental state exam, *NC* normal control, *MCI* mild cognitive impairment, *AD* Alzheimer’s Disease

### An interaction between CSF C3 and APOE ε4 predicts CSF amyloid and CSF ptau

**Fig. 1:**
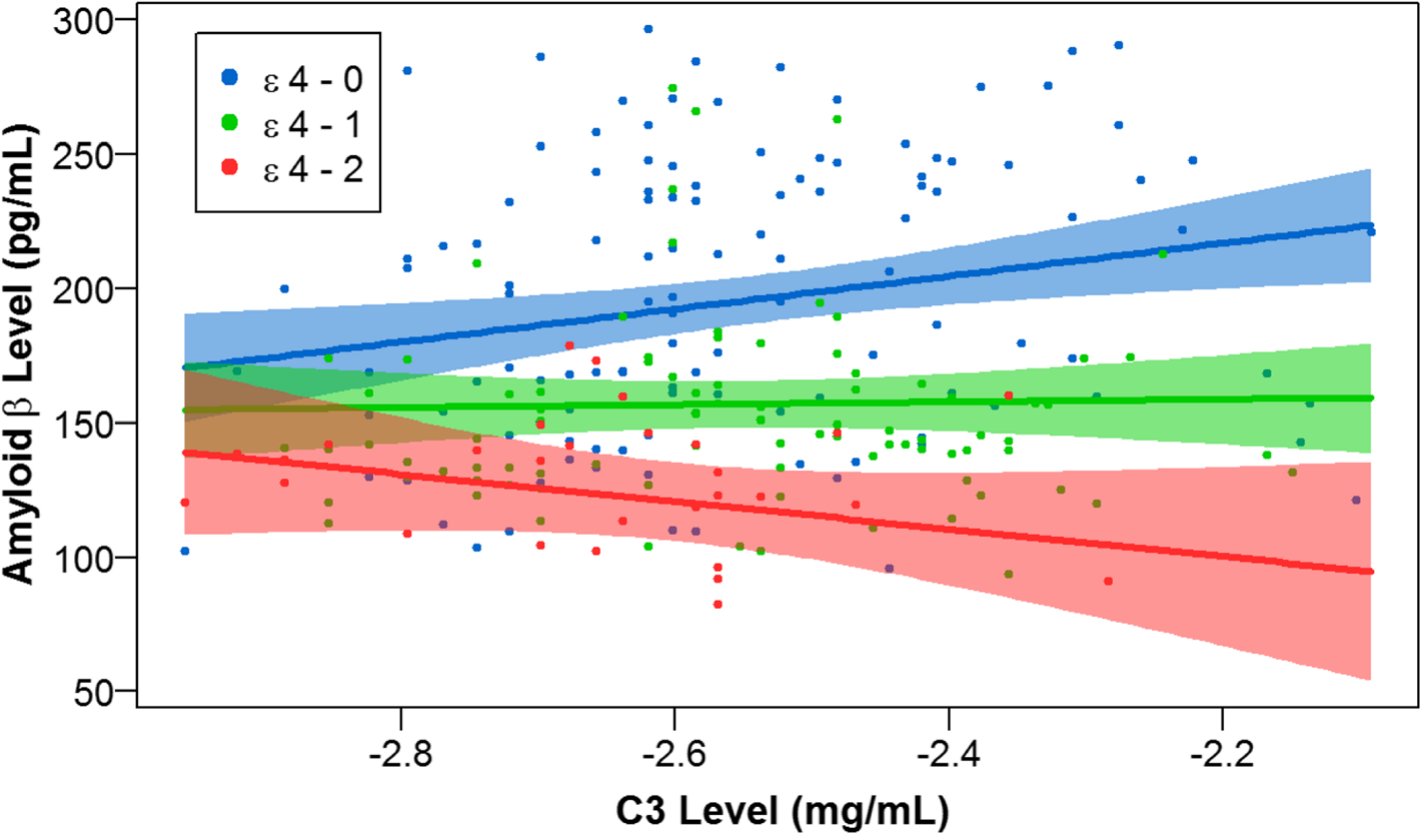
CSF amyloid versus CSF C3 by *APOE* ε4 dosage. CSF amyloid levels are plotted against CSF C3 levels by *APOE* ε4 dosage. C3 levels are quality controlled and transformed as described in [26]. Non-carriers of the *APOE* ε4 allele (*blue*) have the highest level of amyloid in CSF, reflecting lower levels of amyloid pathology in the brain, and display a positive slope. Carriers of one copy of the *APOE* ε4 allele (*green*) have intermediate levels of amyloid and display an approximately flat slope. Carriers of two copies of the *APOE* ε4 allele (*red*) have the lowest levels of CSF amyloid, suggesting the highest levels of amyloid pathology in the brain, and display a negative slope. The plotted points are partial residuals with 95 % confidence bands provided in shading

We found a significant statistical interaction between CSF C3 and *APOE* ε4 dosage on CSF amyloid levels (β-coefficient = -56.2, standard error (SE) = 23.8, *p*-value = 0.02) suggesting increased intracranial amyloid pathology among individuals with elevated intracranial C3 levels and increased number of *APOE* ε4 alleles (Fig. 1). With this interaction term in the model, we found a main effect of CSF C3 (β-coefficient = 61.3, SE = 21.9, *p*-value = 5.55 × 10^−3^) and *APOE* ε4 dosage (β-coefficient = -182.0, SE = 62.0, *p*-value = 3.69 × 10^−3^).

**Fig. 2:**
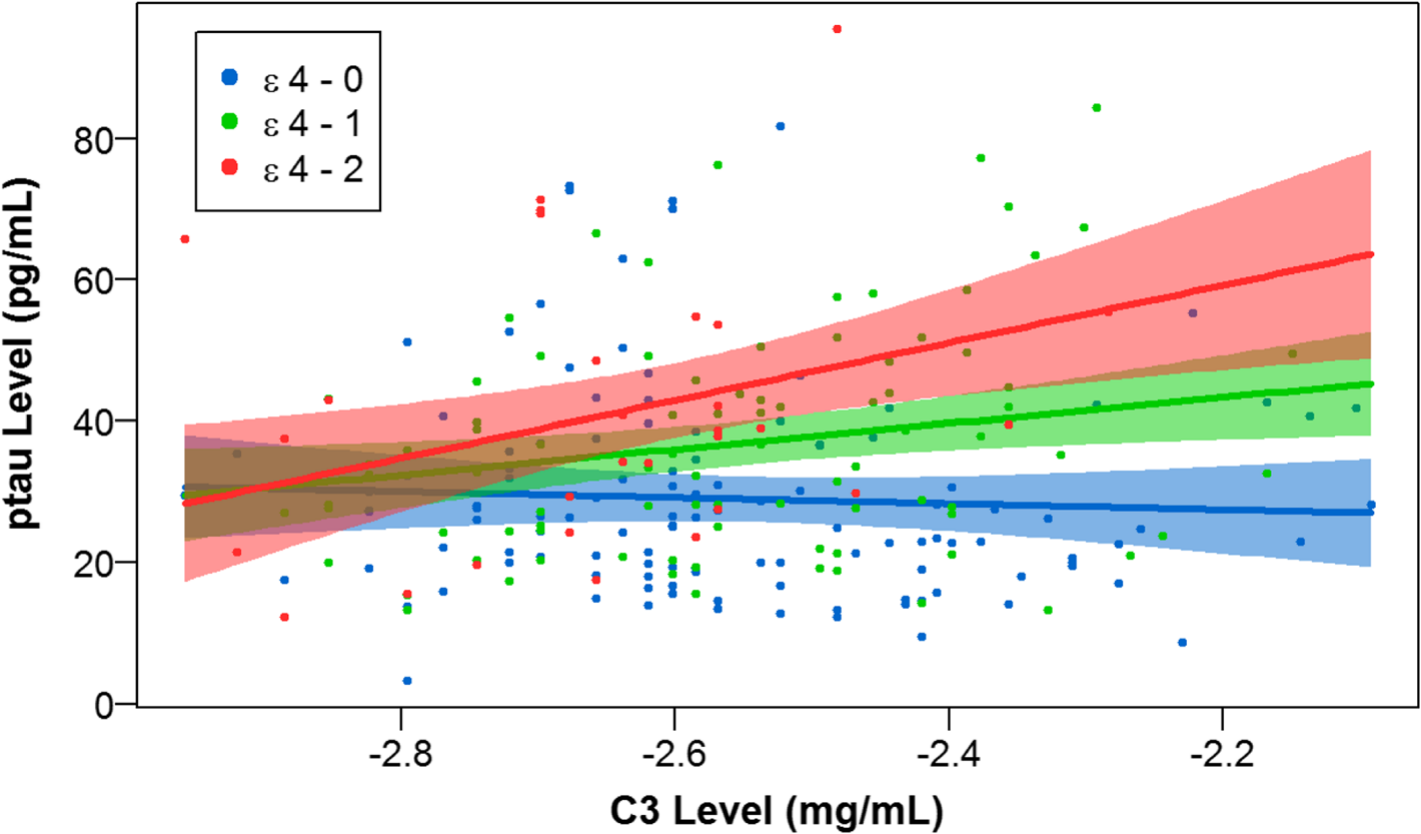
CSF ptau versus CSF C3 by *APOE* ε4 dosage. CSF ptau levels are plotted against CSF C3 levels by *APOE* ε4 dosage. C3 levels are quality controlled and transformed as described in [26]. Non-carriers of the *APOE* ε4 allele (*blue*) have the lowest levels of ptau and display a slightly negative slope. Carriers of one copy of the *APOE* ε4 allele (*green*) have intermediate levels of ptau and display a modestly positive slope. Carriers of two copies of the *APOE* ε4 allele (*red*) have the highest levels of ptau and display a strongly positive slope. The plotted points are partial residuals with 95 % confidence bands provided in shading

Similarly, we found a significant statistical interaction between CSF C3 and *APOE* ε4 dosage on CSF ptau levels (β-coefficient = 22.5, SE = 8.6, *p*-value = 9.44 × 10^−3^) suggesting increased neurofibrillary pathology among individuals with elevated C3 levels and increased number of *APOE* ε4 alleles (Fig. 2). With this interaction term in the model, we found a main effect of *APOE* ε4 dosage (β-coefficient = 65.4, SE = 22.4, *p*-value = 3.86 × 10^−3^) but not CSF C3 (β-coefficient = -4.3, SE = 7.9, *p*-value = 0.59).

### Amyloid statistically mediates the effect of CSF C3 on CSF ptau

**Fig. 3:**
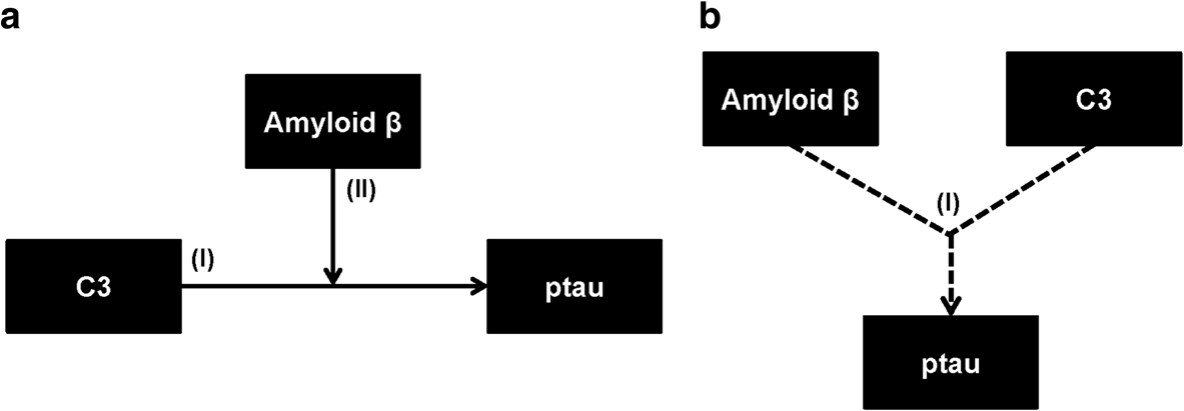
Two possible models for the relationship between C3, amyloid, and ptau. Two possible models for the effects of C3, amyloid β, and *APOE* ε4 on ptau are illustrated as A and B. Solid arrows represent main effects of each variable and dotted arrows represent the interaction effect of two variables, all assessed in multivariate linear models which included age, sex, and CDR-SB score as covariates. Results for the two illustrated models are in Table 2. **a** Model A hypothesizes that C3’s effect on ptau will be mediated by amyloid. Roman numerals represent the following effects: (I) direct effect of C3 to predict ptau and (II) the hypothesized mediating effect of amyloid when added to the model in (I). **b** Model B suggests an alternate relationship whereby C3 and amyloid act synergistically to alter ptau. Roman numeral (I) corresponds to a hypothesized statistical interaction between C3 and amyloid to predict ptau levels

We assessed whether CSF amyloid influences the relationship between CSF C3 and CSF ptau by statistically testing two theoretical models (illustrated in Fig. 3). Model A proposes that amyloid mediates the effect of CSF C3 on CSF ptau. Model B proposes that an interaction between CSF C3 and CSF amyloid alters CSF ptau levels.

Full regression results from Models A and B (shown in Fig. 3) are provided in Table 2. We first tested Model A. We found that CSF C3 did not predict CSF ptau in the baseline model (*p* = 0.35). When CSF amyloid levels were added to the baseline model as a mediating variable, CSF C3 was significantly associated with CSF ptau (*p* = 0.04). Next we assessed Model B. In contrast to Model A, we found that there was no statistical interaction between CSF C3 and CSF amyloid on CSF ptau (*p* = 0.41).

**Table 2:** Results for two proposed models of C3’s effect on ptau in which amyloid mediates the effect of C3 on ptau (Model A) and in which C3 and amyloid act synergistically on ptau (Model B)

Model	Analysis	Outcome	Variable	Estimate ± SE	*P*-value
Model A	I	CSF ptau	Age	−0.32 ± 0.2	0.05
			Sex	−2.41 ± 2.3	0.29
			CDR-SB	3.35 ± 0.6	7.25 × 10^−8^
			C3	6.10 ± 6.5	0.35
	II	CSF ptau	Age	−0.22 ± 0.1	0.14
			Sex	−1.15 ± 2.05	0.58
			CDR-SB	1.67 ± 0.6	5.01 × 10^−3^
			C3	12.11 ± 5.9	0.04
			CSF amyloid	−0.14 ± 0.02	4.97 × 10^−12^
Model B	I	CSF ptau	Age	−0.25 ± 0.15	0.10
			Sex	−1.11 ± 2.1	0.59
			CDR-SB	1.70 ± 0.6	4.47 × 10^−3^
			C3	27.7 ± 19.6	0.16
			CSF amyloid	−0.38 ± 0.3	0.19
			C3:CSF amyloid	−0.09 ± 0.1	0.41

*SE* standard error

An illustration of the two models is provided in Fig. 3. Estimates are β estimates from a multiple regression model which included age, sex, and baseline CDR-SB score as covariates

## Discussion

**Fig. 4:**
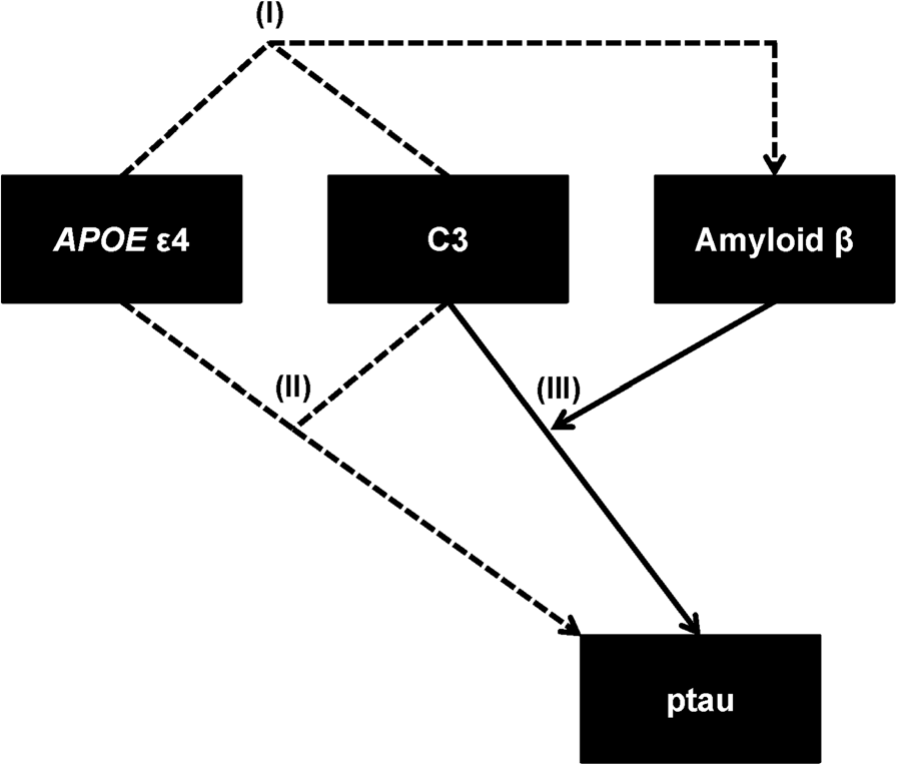
Proposed biological model for the relationships between C3, *APOE* ε4, and amyloid on ptau. A proposed model for the relationships between C3, *APOE* ε4, and amyloid on ptau based on results from our statistical analyses. Solid arrows represent main effects of each variable and dotted arrows represent the interaction effect of two variables, all assessed in multivariate linear models which included age, sex, and CDR-SB score as covariates. Roman numerals represent the following effects: (I) interaction effect of C3 and *APOE* ε4 on amyloid (II) interaction effect of C3 and *APOE* ε4 on ptau, and (III) the effect of C3 on ptau, which is mediated by amyloid

Among individuals with elevated intracranial C3 and increased *APOE* ε4 dosage, we found clinical evidence of elevated intracranial amyloid and neurofibrillary tangle pathology. We also found statistical evidence that amyloid mediates the effect of C3 on ptau pathology. Considered together, our results support a conceptual model of the AD pathogenic cascade where a synergistic relationship between the complement cascade (C3) and *APOE* ε4 results in elevated Alzheimer’s associated pathology and in turn, amyloid further regulates the effect of the complement cascade on ptau pathology (Fig. 4). These results suggest that a combination of inflammatory and pathologic biomarkers may help elucidate the neurodegenerative process underlying clinical AD.

Building on prior genetic evidence implicating a relationship between *APOE* and immune dysfunction, we found signs of elevated Alzheimer’s pathology in the presence of *both APOE* ε4 and elevated complement activation indicating that genetic propensity and inflammation may act synergistically to accelerate amyloid and tau pathology. In the presence of the statistical interaction, we found no effect of C3 on ptau suggesting that *APOE* may play an important role in influencing the relationship between inflammation and tau pathology. Interestingly, even with the interaction term in place, we found a significant effect of C3 on amyloid indicating that the complement cascade could influence amyloid pathology via both *APOE*-dependent and *APOE*-independent mechanisms. Additional experimental work will be required to delineate the precise mechanistic relationship between complement activation, *APOE*, amyloid and tau pathology.

Building on empirical research from prior studies [15, 19], our mediation analyses suggest that amyloid may influence the relationship between C3 and ptau. These results support the hypothesis that amyloid dysmetabolism regulates downstream pathological processes via inflammatory mechanisms. Recent work has shown that inflammatory processes are required for amyloid related synaptotoxicity [15] and other work has shown that amyloid can incite an inflammatory response [27] and specifically activate the complement cascade [1]. Taken together, our findings draw attention to the role of C3 in combination with amyloid and APOE as a potential regulator of downstream pathology.

Our findings suggest that a pro-inflammatory environmental milieu alters AD pathology in conjunction with genotype. This work further illustrates that inflammatory biomarkers along with genetic information may predict increased risk for AD [32] and other neurodegenerative disorders with genetic and immune components [21]. Clinically, this is important because it suggests that modifying specific components of the innate immune system or their downstream signaling pathways may serve as a target, which could ameliorate AD pathology. In addition to amyloid and tau metabolism and clearance, these results, in combination with prior studies [2, 17, 25, 30], underscore the importance of targeting inflammatory (specifically the complement cascade) processes for AD prevention.

Our study benefits from its use of a well-established, thoroughly characterized cohort of AD, MCI and cognitively normal healthy controls. In addition, our study benefits from using CSF, which is a biologically proximate measure of disease status and progression [3, 4]. A limitation of our study is its observational nature, which prevents us from determining a causative relationship. We cannot determine whether C3 causes, results from, or merely correlates with amyloid deposition. The specific biological mechanisms by which C3 and *APOE* ε4 influence ptau and amyloid remain to be determined. Our results will require follow-up in a larger, independent population-based cohort to determine the generalizability of this finding to diverse communities. Finally, our study highlights the need for future longitudinal studies that can directly test the causal effects suggested by our correlative analyses.

## Conclusion

In summary, we found a statistical interaction between elevated C3 and *APOE* ε4 dosage on biomarkers indicative of elevated amyloid and tau pathology. Further, our mediation analyses indicate that C3 is associated with ptau only after adjusting for amyloid suggesting that amyloid may mediate the relationship between inflammation and tau pathology. Our findings underscore the importance of *APOE* ε4 in regulating the putative effects of C3 on both amyloid and ptau. More generally, this study highlights the importance of the innate immune system in modulating levels of the pathological proteins most associated with AD and its relevance as a biomarker and potential therapeutic target.

## Additional file

Additional file 1: ADNI methods. (DOCX 12 kb)
