# Supplementary material for: The relationship between complement factor C3, APOE ε4, amyloid and tau in Alzheimer’s disease
Source: Acta Neuropathol Commun. 2016 Jun 29;4:65. doi: 10.1186/s40478-016-0339-y (PMC4928261; doi:10.1186/s40478-016-0339-y)
Supplement: Additional file 1: — ADNI methods. (DOCX 12 kb) [file 40478_2016_339_MOESM1_ESM.docx]

**ADNI METHODS**

Data used in the preparation of this article were obtained from the Alzheimer’s Disease Neuroimaging Initiative (ADNI) database (adni.loni.usc.edu). The ADNI was launched in 2003 as a public-private partnership, led by Principal Investigator Michael W. Weiner, MD. The primary goal of ADNI has been to test whether serial magnetic resonance imaging (MRI), positron emission tomography (PET), other biological markers, and clinical and neuropsychological assessment can be combined to measure the progression of mild cognitive impairment (MCI) and early Alzheimer’s disease (AD).
